# Discovery of a DNA repair-associated radiosensitivity index for predicting radiotherapy efficacy in breast cancer

**DOI:** 10.3389/fonc.2025.1439516

**Published:** 2025-03-25

**Authors:** Jianguang Lin, Hainan Yang, Rongfu Huang, Tianwen Xu

**Affiliations:** ^1^ Department of Oncology, The Second Affiliated Hospital of Fujian Medical University, Quanzhou, Fujian, China; ^2^ Department of Ultrasound, First Affiliated Hospital of Xiamen University, School of Medicine, Xiamen University, Xiamen, Fujian, China; ^3^ Department of Clinical Laboratory, The Second Affiliated Hospital of Fujian Medical University, Quanzhou, Fujian, China

**Keywords:** breast cancer, DNA repair pathway, radiosensitivity, PD-L1, tumor immune microenvironment

## Abstract

**Purpose:**

Radiotherapy is a cornerstone of breast cancer (BRCA) treatment. Accurately predicting tumor radiosensitivity is critical for optimizing therapeutic outcomes and personalizing treatment strategies. DNA repair pathways are key determinants of radiotherapy response. Thus, we aimed to develop a novel DNA repair-related radiosensitivity model and to identify potential targets for enhancing radiotherapy efficacy.

**Methods:**

A retrospective study was conducted using data from 942 BRCA patients from TCGA database. A radiosensitivity model, comprising a radiosensitivity index, was developed using LASSO regression analysis. Patients were stratified into radiosensitive (RS) and radioresistant (RR) groups based on their radiosensitivity index (RSI). Associations between the RSI, clinicopathological parameters, and PD-L1 status were analyzed. The CIBERSORT and ESTIMATE algorithms were employed to characterize the immune landscape of the tumor microenvironment. The Tumor Immune Dysfunction and Exclusion (TIDE) algorithm and pRRophetic platform were used to predict treatment responses. Key genes identified in the radiosensitivity model were further validated using *in vitro* qRT-PCR experiments.

**Results:**

We successfully constructed a radiosensitivity index incorporating 10 DNA repair-related genes. Patients in the RS group exhibited significantly better prognosis compared to the RR group, but this benefit was limited to those receiving radiotherapy. This survival benefit associated with the radiosensitivity signature was absent in patients who did not receive radiotherapy. The RS group displayed a distinct molecular profile characterized by enrichment of TGF-β signaling and protein secretion pathways, potentially contributing to enhanced radiosensitivity. Furthermore, the RS group exhibited increased infiltration of immune cells. Notably, the RS-PD-L1-high subgroup demonstrated the most favorable survival outcomes and highest immune cell infiltration, highlighting their potential responsiveness to immunotherapy. In addition, the RR group exhibited a distinct profile characterized by enrichment of DNA repair pathways and a heightened sensitivity to CDK and HER2 inhibitors. Conversely, this group displayed resistance to DNA-damaging drugs. These findings were supported by *in vitro* experiments using MCF-7 and radioresistant MCF-7/IR cell lines, confirming differential expression of key radiosensitivity index genes.

**Conclusion:**

In conclusion, we established a radiosensitivity model for predicting radiotherapy benefit in breast cancer. Our study reveals a strong association between radiosensitivity, enhanced antitumor immunity, and potential immunotherapy benefit, particularly within the RS-PD-L1-high subgroup.

## 1 Introduction

Breast cancer (BRCA) remains a significant global health challenge. According to the latest statistics from *Cancer Statistics, 2024* breast cancer is the most commonly diagnosed cancer in women worldwide, accounting for an estimated 32% of all new cancer diagnoses (1). While the 5-year relative survival rate for localized breast cancer is high, it decreases significantly to 31% for metastatic disease, highlighting the urgent need for improved prognostic and predictive biomarkers to guide treatment decisions and improve patient outcomes (2). Cancer therapy has evolved significantly, encompassing surgery, chemotherapy, radiation therapy, targeted therapy, and immunotherapy (3). Radiotherapy is commonly employed across various treatment modalities for BRCA patients at different stages, offering evident long-term advantages in terms of both locoregional control and reduced mortality (4–6). Nevertheless, the efficacy of radiotherapy, the enduring nature of its response, and the emergence of radio-resistance remain significant obstacles in BRCA treatment (7, 8). The molecular mechanisms underlying tumor sensitivity to radiotherapy are intricate, and currently, there exists no definitive biomarker to assess radiation response (9). However, recent studies have identified several promising candidates, particularly those involved in DNA damage and repair mechanisms (10, 11), warranting further investigation into their potential clinical utility in predicting radiotherapy response.

In the epoch of precision medicine, the investigation into radiosensitivity at the genomic level has attracted significant attention. Various groups have independently developed genomic assays that have the capability to predict radiation response with different types of cancer (12–14). Through the utilization of high-throughput molecular profiling, a variety of prognostic gene signatures have been formulated to categorize patients based on risk and identify those most likely to experience favorable outcomes from radiotherapy (15, 16). For example, the radiosensitivity index (RSI), a rank-based signature comprising ten genes, has been developed to predict the sensitivity of 48 cancer cell lines to radiation. This index has undergone independent validation in multiple cancer types, including BRCA (17). Additionally, a distinct radiosensitivity 31-gene signature, derived from microarray data sourced from NCI-60 cancer cells, has emerged as a significant determinant for prognosticating clinical outcomes in patients with BRCA following radiotherapy, and may be used for selecting patients who will derive benefits from radiotherapy combined with immunotherapy (18). Furthermore, a multitude of researchers are working towards predicting treatment responses when radiotherapy is combined with other therapeutic modalities, such as immunotherapy and chemotherapy (19).

DNA damage is the primary and intrinsic factor that plays a critical role in the response to radiation (20, 21). The fundamental determinant of cellular radiosensitivity lies in the ability of cells to repair lethal DNA double-strand breaks (22). In recent decades, the targeting of signaling pathways involved in DNA damage repair (DDR) has emerged as an alluring strategy for augmenting the cancer response to radiation and surmounting tumor radio-resistance (23–25). Previous investigations have demonstrated the significant roles played by DNA damage repair mechanisms in the occurrence, progression, and therapeutic efficacy of BRCA (26, 27). Notably, approximately 50%-80% of hereditary BRCA cases involve mutations in BRCA1 or BRCA2, both of which are intricately involved in the DNA damage response (28). Hence, it becomes imperative to explore the correlation between DNA damage repair signaling pathways and the response to radiotherapy. However, no study has yet delved into the examination of the potential of DNA repair-related signatures as biomarkers for BRCA.

In our current study, we aimed to explore the potential of a DNA repair-related radiosensitivity index as a predictive biomarker for response to radiotherapy. We developed a novel index that incorporates DNA repair-related genes and assessed its ability to identify patients who would benefit from radiotherapy. By stratifying BRCA patients based on this radiosensitivity index, we observed significant differences in therapeutic sensitivity to radiotherapy, functional enrichment pathways, and tumor immune microenvironment landscapes. This suggests that the index can provide valuable insights into the diversity of radiosensitivity among BRCA patients. Furthermore, the radiosensitivity model we developed in this study has the potential to enhance our understanding of the optimal combination of drugs with radiotherapy.

## 2 Methods

### 2.1 Data collection and processing

We obtained the RNA-sequencing (RNA-seq) data and clinical information of BRCA specimens from The Cancer Genome Atlas (TCGA) (https://portal.gdc.cancer.gov/) and The Molecular Taxonomy of Breast Cancer International Consortium (METABRIC) database (http://www.cbioportal.org/) (29, 30). A total of 942 patients were selected for further analysis based on the availability of both RNA-seq and clinical data. RNA-seq data normalization was performed using the fragments per kilobase of exon model per million mapped fragments (FPKM) method, and these values were subsequently converted to transcripts per million (TPM) to improve comparability of gene expression levels between samples. A list of 219 human DNA repair genes (DRGs) from published resources (https://www.mdanderson.org/documents/Labs/Wood-Laboratory/human-dna-repair-genes.html) (31, 32) were selected to screen the gene expression profiles and establish a radiosensitivity model.

### 2.2 Construction of the DNA repair-related radiosensitivity model

Firstly, we employed univariate Cox regression analysis to identify individual DRGs that exhibited significant associations with overall survival (OS) in radiotherapy patients (significance threshold: p < 0.05) but not in non-radiotherapy patients. All radiotherapy patients within the TCGA dataset were then used for radiosensitivity model construction. Subsequently, we utilized lasso penalized regression analysis, leveraging the “glmnet (version 4.1-7) “ package, to construct a radiosensitivity model based on the identified DRGs (33, 34). This model aimed to quantify the radiosensitivity of patients. By employing the formula: radiosensitivity index = (Expression gene 1 × Coefficient gene 1) + (Expression gene 2 × Coefficient gene 2) + · · · + (Expression gene n × Coefficient gene n), we generated a scoring system that predicts the survival of patients. Each patient within the TCGA-BRCA dataset was assigned a radiosensitivity index using this formula. Based on the median value of the radiosensitivity index, the radiotherapy patients were further categorized into two groups: the radiosensitive (RS) group and the radioresistant (RR) group. The RS group represented patients who exhibited improved survival after receiving radiotherapy compared to those who did not receive radiotherapy. It’s important to note that the survival rate of the RS group was not superior to that of the RR group when neither group received radiotherapy.

### 2.3 Mutation analysis of DRGs

TCGA mutation annotation format (MAF) data of BRCA was downloaded from TCGA database (https://portal.gdc.cancer.gov/). The “maftools (version 2.10.05)” package was utilized to generate the mutation frequency and waterfall plot for 10 DRGs in BRCA patients (35). Additionally, the “circlize (version 0.4.15)” package was employed to depict the locations of these 10 DRGs on the 23 chromosomes (36). Copy number variation (CNV) data for the TCGA BRCA cohort was obtained from the TCGA GDC Data Portal. The CNV analyses were performed using gene-level data generated by GISTIC 2.0, which allows for high-resolution detection of genomic alterations. In order to visualize the CNV values, a threshold of 0.3 was set. The frequency of CNV was then represented using a Cleveland dot plot, which was created using the “ggpubr (version 0.6.0)” package.

### 2.4 Functional enrichment analysis and gene set variation analysis

To assess the variation in pathway activity between the RS and RR groups, we utilized the GSVA method using the “GSVA (version 1.42.0)” package. For this analysis, we retrieved the ‘hallmark gene sets.v2023.1.Hs.symbols.gmt’ gene set from the Molecular Signatures Database (MSigDB) and selected it as the background gene set. To compare the differential activity of the hallmark pathways between two groups, we employed the R package “limma (version 3.50.3)” (37). Furthermore, we conducted Gene Set Enrichment Analysis (GSEA) to identify the biological processes (BP), molecular functions (MF), and cellular components (CC) associated with the RS and RR groups. The statistical significance for this analysis was determined using an adjusted p-value threshold of less than 0.05.

### 2.5 Quantification of immune infiltration

CIBERSORT analysis was performed using the LM22 signature matrix, which represents the gene expression profiles of 22 human immune cell subtypes (38). The algorithm was run with 1000 permutations, and estimates with a p-value < 0.05 were considered statistically significant and included in downstream analyses (39). Prior to deconvolution, the CIBERSORT tool internally normalizes the gene expression data using quantile normalization. Subsequently, we employed the “reshape2 (version 1.4.4)” packages to visualize the relationship among the risk score, DRGs, and immune cell populations. The ESTIMATE algorithm was chosen to infer the fraction of stromal and immune cells in tumor samples based on gene expression data (40). The ESTIMATE algorithm was applied using the default parameters in the “estimate (version 1.0.13)” R package. The gene expression data was used as input to calculate the immune score, stromal score, and tumor purity for each sample.

### 2.6 Predicting individual sensitivity to immunotherapy, chemotherapeutic and targeted agents

The Tumor Immune Dysfunction and Exclusion (TIDE) algorithm was utilized to quantify tumor immunogenicity and immune evasion, providing valuable insights into the efficacy of immunotherapy for BRCA patients (41). The TIDE algorithm was implemented using the online tool available at https://tide.dfci.harvard.edu/. The gene expression data was uploaded, and the analysis was performed using the default parameters for breast cancer. To assess the predictive accuracy of the model for responses to chemotherapy and targeted therapy, we employed the R package pRRophetic (version 0.5) to calculate the half-maximal inhibitory concentration (IC50) of samples in both the RS and RR groups using ridge regression (42). The analysis was performed using the gene expression data and the GDSC training dataset. The mean IC50 value was calculated for each drug within the RS and RR groups. Subsequently, a Wilcoxon signed-rank test was conducted to compare the IC50 values between the RS and RR groups for further analysis.

### 2.7 Establishment of radioresistant BRCA cells

For *in vitro* validation, we selected MCF-7 and MCF-7/IR cell lines to represent the RS and RR groups, respectively. MCF-7 cells are a commonly used breast cancer cell line, while MCF-7/IR cells are a radioresistant variant derived from MCF-7 cells through fractionated irradiation. Both cell lines were cultured in Minimum Essential Medium supplemented with 10% fetal bovine serum (Corning, United States) and 1% antibiotics (Gibco-BRL, United States). The establishment of MCF-7/IR cells involved subjecting MCF-7 cells to fractionated ionizing radiation with a total dose of 60 Gy of γ-irradiation (2 Gy per fraction, administered five times per week for a total of 6 weeks). As for the parental MCF-7 cells, they underwent a similar procedure but were sham-irradiated, serving as the control group for comparison. Cell proliferation assays were performed to evaluate the radio-resistance of MCF-7/IR cells after exposure to ionizing radiation.

### 2.8 Cell proliferation assays and quantitative real-time polymerase chain reaction

Cell viability was assessed using the CCK-8 assay according to the manufacturer’s instructions. The cells were seeded in 96-well plates and incubated. After 24 hours, the cells were exposed to irradiation doses of 2, 4, or 8 Gy. Following the irradiation, 10 μl of CCK-8 solution (Dojindo, Kumamoto, Japan) was added to each well, and the cells were further incubated for three hours. The optical density of the solution was measured at 450 nm using a microplate reader.

RNA extraction was performed using Trizol reagent, following the manufacturer’s protocol (Invitrogen, San Diego, CA, USA). The concentration of the extracted RNA was determined using a NanoDrop 2000 spectrophotometer (ThermoFisher, USA). For cDNA synthesis, the transcriptor First Strand cDNA Synthesis Kit (Roche, Germany) was used with the extracted RNA as the template. Quantitative RT-qPCR was carried out using the SYBR Prime Script RT–PCR Kit (Invitrogen, USA), and the primer sequences can be found in **Supplementary Table S1**. All experiments were conducted in triplicate for each independent experiment to ensure the reproducibility of the results. Ct values were normalized to the genomic mean of the internal control gene, GAPDH. The relative expression levels were calculated using the 2^-ΔΔCT^ method. The specific formula used for calculating relative gene expression was: 2^^-[ΔCt(target gene) - ΔCt(GAPDH)]^, where ΔCt = Ct(MCF-7/IR) - Ct(MCF-7). Statistical significance was determined using a two-tailed Student’s t-test to compare the relative expression levels between MCF-7 and MCF-7/IR cells.

### 2.9 Statistical analysis

Statistical analysis was conducted using R software version 4.1.3. The student’s t-test was used for comparing normally distributed data between two groups, whereas the Chi-square test was utilized to compare categorical and pairwise characteristics across distinct groups. For determination of statistically significant distinctions between two groups, the Mann-Whitney U test was applied, and the Kruskal-Wallis test was employed for evaluating statistically significant disparities amidst multiple independent groups. Pearson’s correlation test was utilized to examine associations between variables that exhibited normal distribution, whereas Spearman’s correlation test was employed for evaluating relationships between variables that deviated from normal distribution. Survival disparities among two or more groups were scrutinized using the Kaplan-Meier method and log-rank test. To account for multiple hypothesis testing, we applied False Discovery Rate (FDR) correction using the Benjamini-Hochberg method on the p-values obtained from our analyses. An FDR threshold of 0.05 was used to determine statistical significance.

## 3 Results

### 3.1 Construction of a radiosensitivity model based on DNA repair-related genes

**Figure 1:**
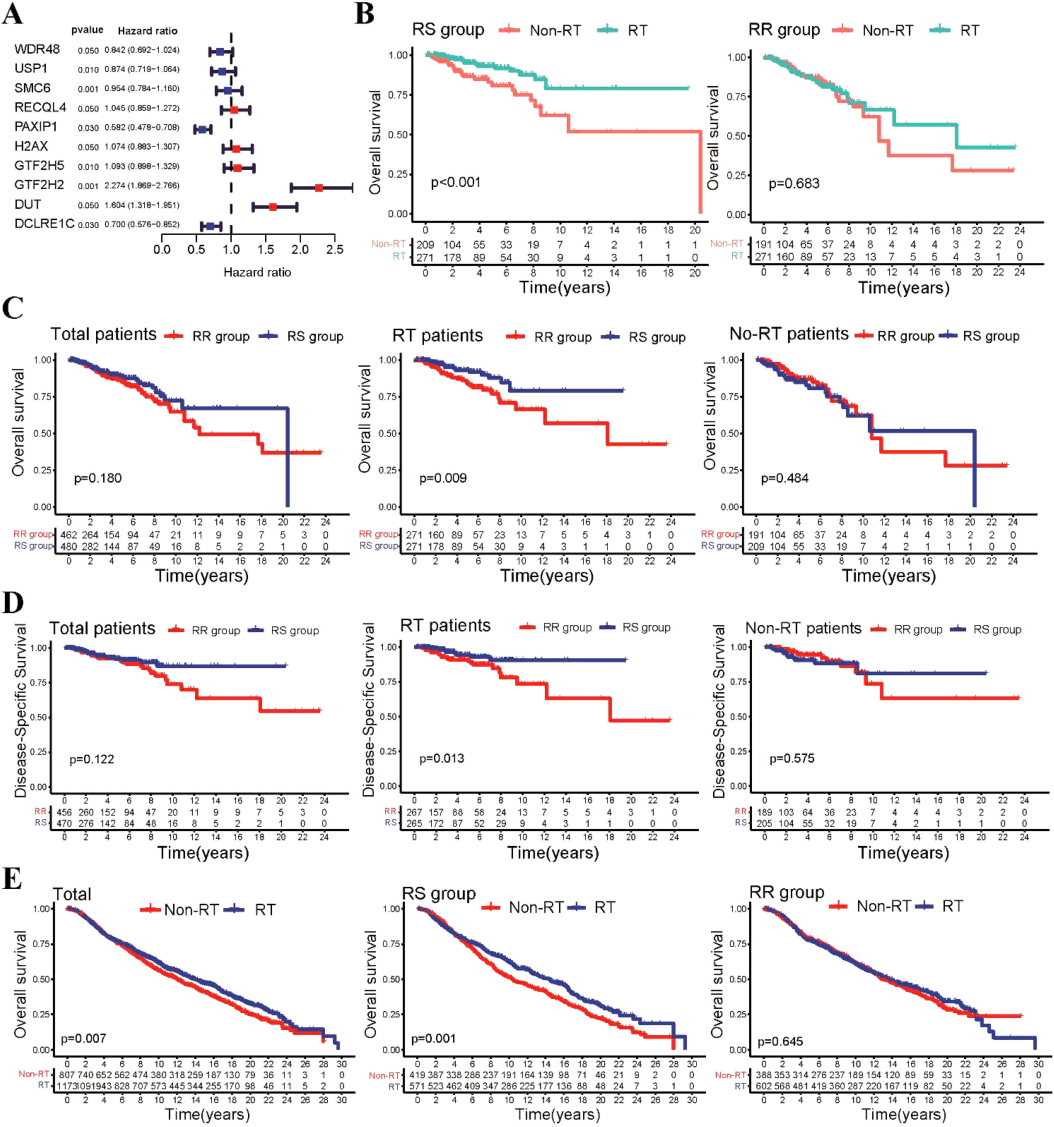
Construction of the repair-related radiosensitivity index in the TCGA dataset. **(A)** Forest plot showing hazard ratios (HR) for genes in the radiosensitivity signature. **(B)** Kaplan-Meier plot illustrating the OS outcomes of patients who received radiotherapy compared to non-radiotherapy patients within the RS and RR groups. **(C)** Kaplan-Meier survival curves depicting the OS outcomes of patients in the RS and RR groups within the radiotherapy and non-radiotherapy patients. **(D)** Kaplan-Meier survival curves showing the DSS outcomes of patients in the RS and RR groups within the radiotherapy patient and non-radiotherapy patients. **(E)** Kaplan-Meier curves illustrating OS for patients in the METABRIC cohort.

Firstly, we conducted univariate Cox regression analysis to identify prognostic DNA repair-related genes in both radiotherapy (RT) and non-radiotherapy (Non-RT) patients from the TCGA-BRCA dataset. We found that 15 DNA repair-related genes were significantly associated with OS in radiotherapy patients, but not in non-radiotherapy patients (**Supplementary Figure S1A**). Subsequently, we employed LASSO penalized regression analysis to construct a radiosensitivity model specifically for radiotherapy patients (**Supplementary Figure S1B**). Through this analysis, we identified 10 genes that were crucial for establishing the radiosensitivity model (**Figure 1A**). Using the median radiosensitivity index of the radiosensitivity model in radiotherapy patients, we classified entire TCGA-BRCA patients into RS and RR groups. The formula for this model is presented in (**Supplementary Table S2**). The Kaplan-Meier survival curve demonstrated that patients who received radiotherapy exhibited significantly improved OS compared to non-radiotherapy patients in the RS group, while there was no significant difference in OS rate was observed between radiotherapy and non-radiotherapy patients in the RR group (**Figure 1B**).

Considering the potential clinical differences between radiotherapy and non-radiotherapy patients that may influence patient survival, we performed additional analyses to examine the survival disparities between the RS and RR groups within the entire BRCA patient cohort. Our findings revealed that in radiotherapy patients, those in the RS group exhibited significantly improved OS compared to those in the RR group. However, no significant differences in OS rates were observed between the RS and RR groups in non-radiotherapy patients (**Figure 1C**). Furthermore, we also investigated disease-specific survival (DSS) and disease-free interval (DFI) and found that radiotherapy patients in the RS group demonstrated better DSS and DFI outcomes compared to those in the RR group (**Figure 1D**, **Supplementary Figure S1C**). To further assess the generalizability of our findings, we validated our radiosensitivity index using an independent cohort from the METABRIC database. Using the same formula established in the TCGA-BRCA analysis, METABRIC patients were classified into RS and RR groups. Survival analysis revealed a significant improvement in OS for radiotherapy patients within the RS group compared to those who did not receive radiotherapy (**Figure 1E**). Collectively, these results suggest that the radiosensitivity index can potentially serve as a valuable radiosensitivity signature for predicting the response to radiotherapy in BRCA patients.

### 3.2 Correlation between radiosensitivity, tumor characteristics, and clinicopathological factors

**Figure 2:**
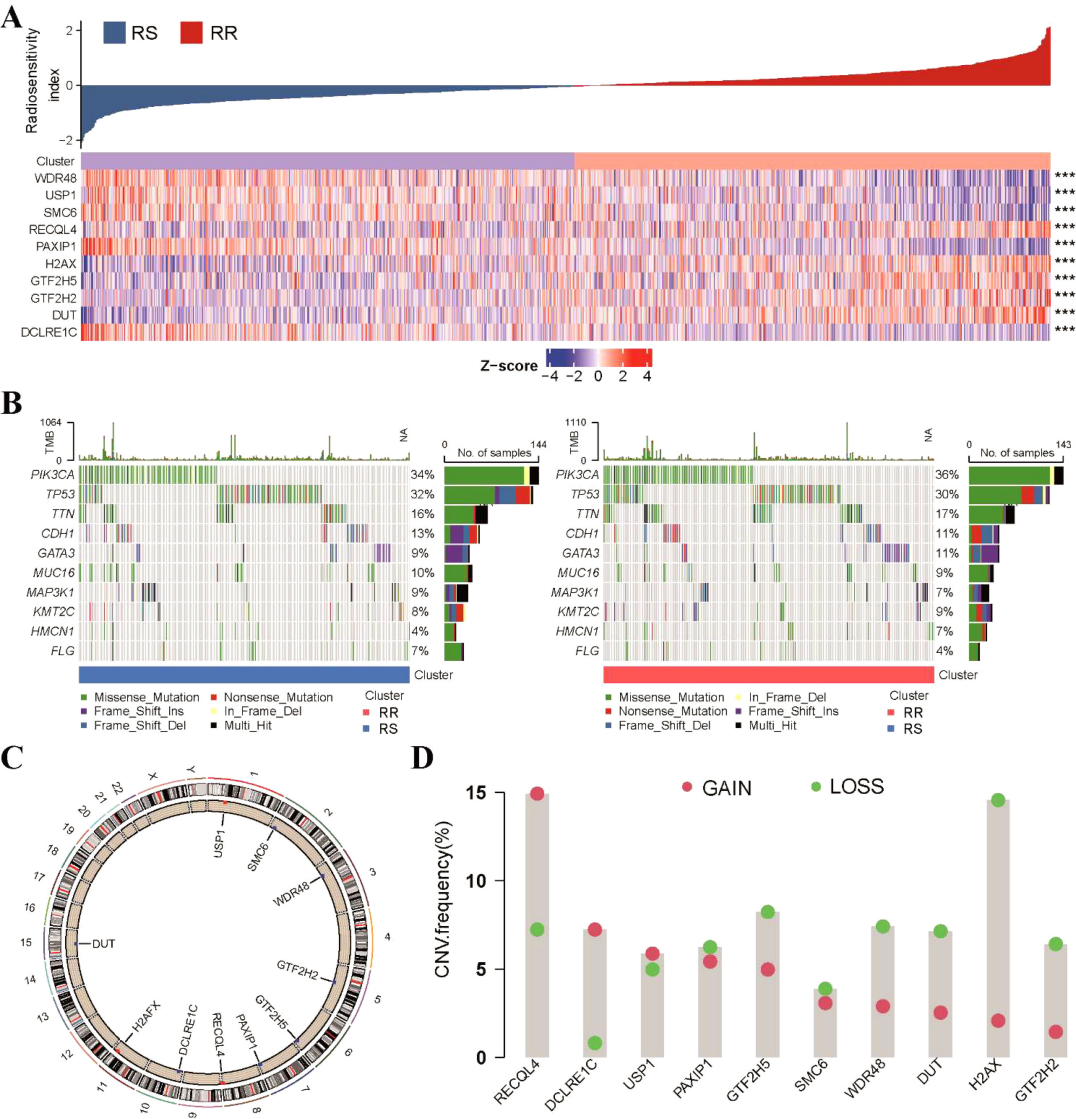
Genetic variation analysis of the 10-gene radiosensitivity index in the TCGA-BRCA dataset. **(A)** The heatmap represented the expression profile of the 10-gene radiosensitivity index in relation to the RS and RR groups. **(B)** Waterfall plots displayed the top 10 frequently mutated genes within the two radiosensitivity groups. **(C)** The chromosomal location of CNV affecting the 10 repair-related genes across the 23 chromosomes. **(D)** The frequency of CNV alterations in the 10 repair-related genes within the TCGA cohort. The column heights indicate the proportions of gain or loss variations observed. ***p<0.001.

We then explored the expression profile of the 10-gene radiosensitivity index in the TCGA-BRCA dataset. Differential expression analysis revealed significant differences in the expression levels of these 10 genes between the RS and RR groups (**Figure 2A**). Genetic analysis at the level of tumor mutational burden (TMB) and stemness index (mRNAsi) showed no significant differences between the RS and RR groups (**Supplementary Figure S2A**). A comprehensive mutational profile was also generated, depicting the top 10 mutated genes. Among various mutation types, missense mutations were the most frequently observed. Notably, PIK3CA and TP53 were the genes most frequently affected by mutations (**Figure 2B**). Furthermore, the chromosomal location of CNV affecting these 10 DNA repair-related genes was illustrated (**Figure 2C**). However, the occurrence of CNV alterations was not uniform across all the genes in the 10-gene radiosensitivity index. Specifically, RECQL4 and DCLRE1C exhibited copy number amplification, while H2AX, GTF2H5, RECQL4, WDR48, and DUT displayed significant CNV deletions (**Figure 2D**). These findings suggest that CNV alterations in these repair-related genes could potentially contribute to the observed abnormal gene expression patterns.

**Figure 3:**
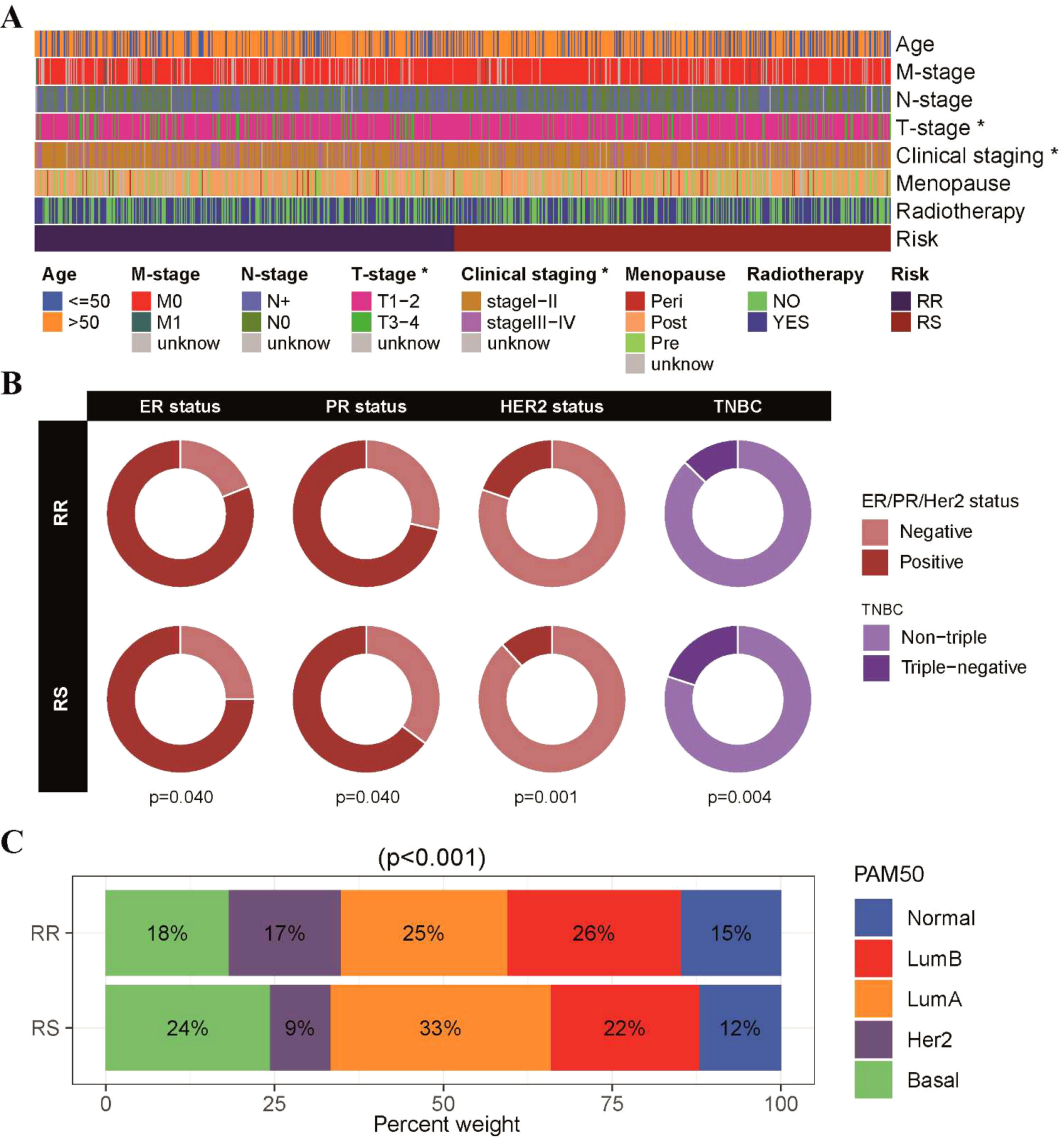
Correlation between radiosensitivity models and pathological features in the TCGA-BRCA dataset. **(A)** An overview of the relationship between the radiosensitivity cluster and clinicopathological parameters in patients with BRCA. **(B)** The doughnut chart visually represented the association between radiosensitivity cluster and the status of hormone receptors in breast cancer. **(C)** The barplot illustrated the proportions of radiosensitivity cluster observed across different PAM50 subtypes. *p<0.05.

Next, we conducted an analysis to investigate the relationships between clinicopathological parameters and the radiosensitivity cluster in our study (**Supplementary Table S3**). A comparison of clinical factors between the two clusters revealed differences in T stage and cancer staging distribution. Specifically, the RS group had a higher proportion of patients with T1-T2 stage tumors, while the RR group had a higher proportion of patients with T3-T4 stage tumors. This observation was further supported by the finding that the RS group had a higher proportion of patients with early-stage BRCA (stage I and stage II), as depicted in **Figure 3A** and **Supplementary Figure S2B**. Additionally, we examined the association between radiosensitivity and the status of hormone receptors in BRCA. Our analysis revealed a higher prevalence of ER/PR-positive and HER2-positive tumors in the RR group, while a higher prevalence of triple-negative BRCA was observed in the RS group (**Figure 3B**). Furthermore, when considering the PAM50 subtype classification, we observed distinct patterns in the distribution of patients within the radiosensitivity groups. Specifically, the RR group exhibited a higher proportion of patients with normal-like, luminal-B, and HER2-type tumors, whereas the RS group showed a higher proportion of patients with basal and luminal-A subtype tumors (**Figure 3C**).

### 3.3 Functional enrichment analysis of radiosensitivity clusters in BRCA

**Figure 4:**
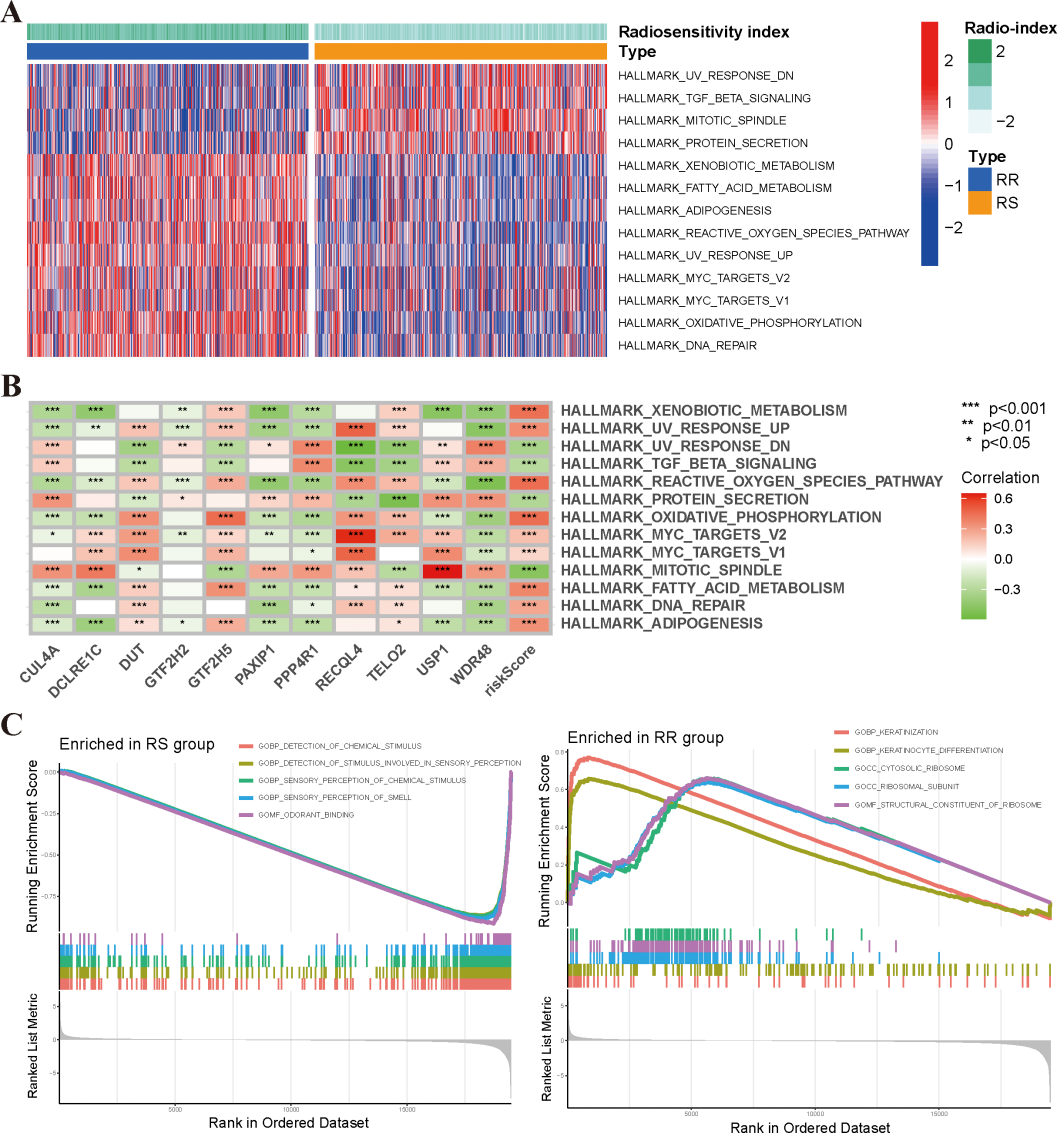
Functional enrichment analysis of the radiosensitivity cluster in BRCA. **(A)** The heatmap illustrated the enrichment scores of the top differentially enriched hallmark pathways, as determined by GSVA analysis, between the RS and RR groups. **(B)** Correlation analysis between the 10-gene radiosensitivity index and hallmark molecular pathways in BRCA. **(C)** GSEA analysis of biological functions comparing the RS and RR groups. *p<0.05, **p<0.01, ***p<0.001.

In order to gain insights into the underlying molecular pathways associated with radiosensitivity in BRCA, we conducted GSVA enrichment analysis to assess the differential activation of hallmark pathways between the radiosensitivity clusters. Our findings revealed distinct pathway associations for the RS and RR groups. Specifically, the RS group exhibited a strong association with the TGF-β Signaling and protein secretion pathway, while the RR group demonstrated predominant correlations with pathways involved in DNA repair, oxidative phosphorylation, and UV response, indicating their potential involvement in radio-resistance mechanisms (**Figure 4A**). We further analyzed the relationship between the expression of the 10-gene radiosensitivity index and these molecular pathways in BRCA (**Figure 4B**). Additionally, we employed GSEA to explore pathway enrichment differences between the RS and RR groups. Notably, the RS group exhibited enrichment primarily in biological processes related to the detection of chemical stimuli and sensory perception. Conversely, the RR group displayed enrichment in pathways associated with keratinocyte differentiation and keratinization (**Figure 4C**).

### 3.4 Correlation between radiosensitivity model and tumor immune infiltration status

**Figure 5:**
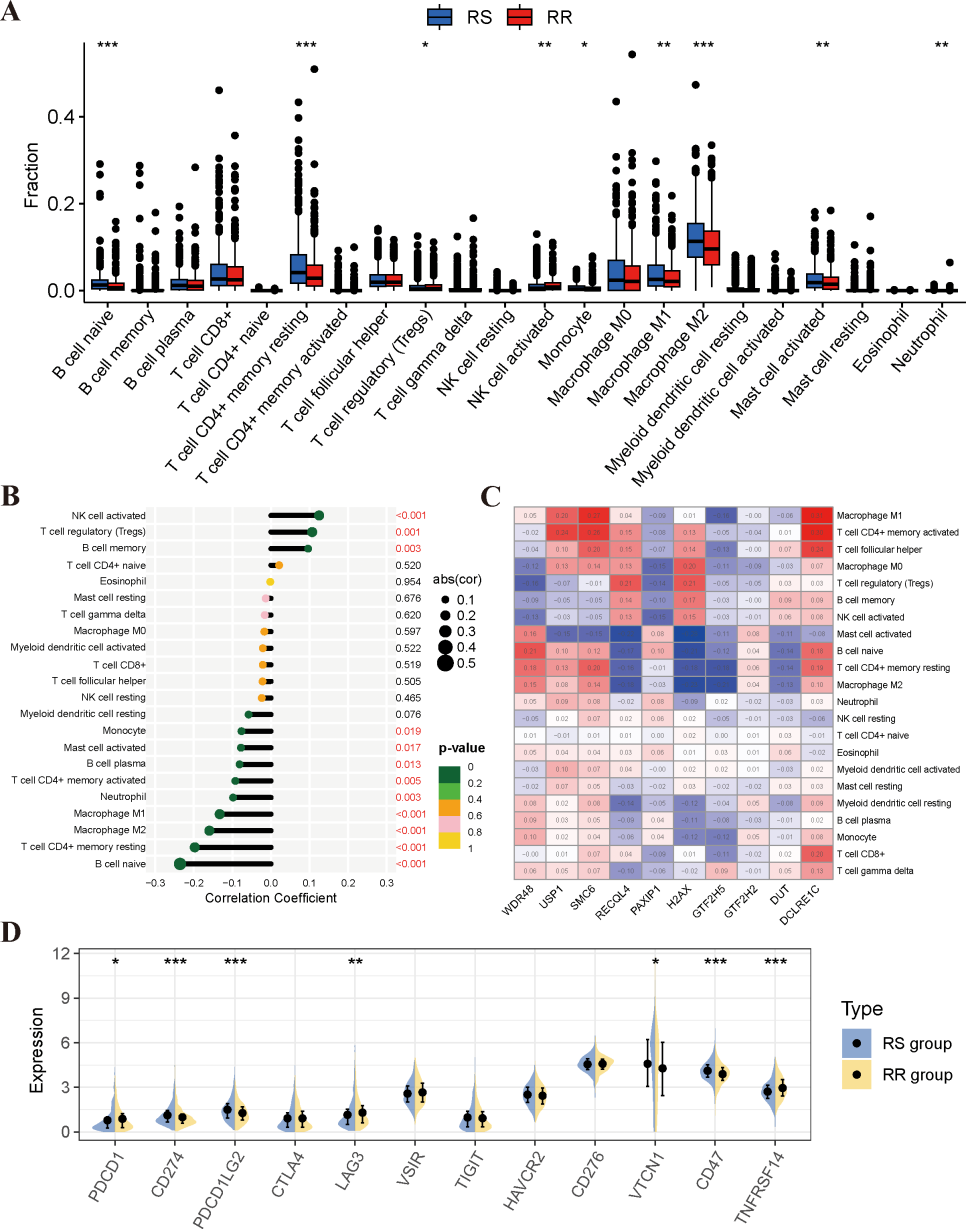
Analysis of immune infiltrating cell profiles in RS and RR groups. **(A)** Variations in the proportion of immune-infiltrating cells among the RS and RR groups. **(B)** Analysis of the correlation between immune-infiltrating cells and the radiosensitivity model. **(C)** Examination of the correlation between immune cells and 10 genes in the radiosensitivity index. **(D)** The expression levels of key immune checkpoint genes were examined between the RS and RR groups. *p<0.05, **p<0.01, ***p<0.001.

There has been a growing body of research highlighting the pivotal role of the tumor immune microenvironment (TIME) in determining the response to radiotherapy. In order to further investigate the relationship between the radiosensitivity index and the TIME, the CIBERSORT algorithm and ESTIMATE analyses were used to assess the differences in the immune landscape between two groups. The results of the CIBERSORT analysis indicated that the RS group displayed higher proportions of naïve B cells, resting CD4 T memory cells, M1/M2 macrophages, and activated mast cells. Conversely, activated NK cells and regulatory T cells were found to be higher in the RR group (**Figure 5A**). Furthermore, correlation analyses revealed a significant negative correlation between the radiosensitivity index and the aforementioned immune cells (**Figure 5B**). Additionally, **Figure 5C** illustrates the associations between the expression levels of the 10-gene radiosensitivity index and the presence of 22 tumor-infiltrating immune cells in BRCA. The results from the ESTIMATE analysis revealed that there were no significant differences in immune score, stromal score, and tumor purity between the RS and RR groups (**Supplementary Figure S3**). Finally, we assessed the expression of immune checkpoint genes, including PD-1, PD-L1, PD-L2, LAG-3, TIM-3, TIGIT, B7-H4, HVEM, and CD47, in the two groups. Our findings indicated that the RS group exhibited higher expression levels of PD-L1, PD-L2, B7-H4, and CD47 compared to the RR group. Conversely, the expression levels of PD-1, LAG-3, and HVEM were higher in the RR group (**Figure 5D**).

### 3.5 Correlation between the radiosensitivity model and PD-L1 status

**Figure 6:**
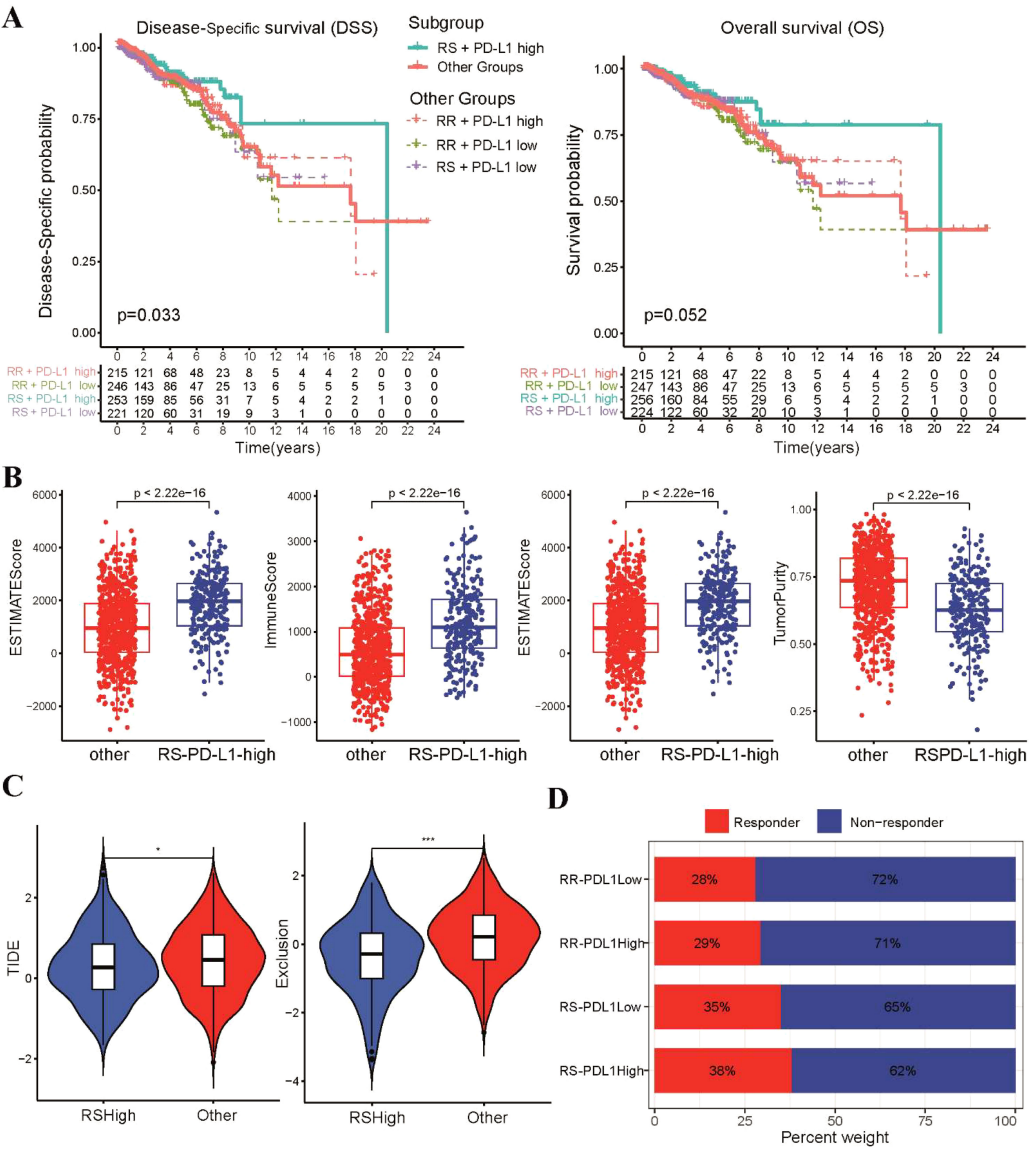
Analysis of immune infiltrating status according to radiosensitivity and PD-L1 status. **(A)** The Kaplan-Meier survival curves of DSS and OS between the RS-PD-L1-high and other groups. **(B)** The levels of infiltration for estimate scores, stromal scores, immune scores, and tumor purity between the two groups were compared using scatter plot. **(C)** The violin plots depict the TIDE and T cell exclusion scores between the two groups. **(D)** The stacked bar chart illustrates the proportion of patients classified as responders and non-responders to immunotherapy within four subgroups *p<0.05, ***p<0.001.

Given the significance of PD-1/PD-L1 in BRCA patients as reported by recent studies (43, 44), we further explored the relationship between radiosensitivity model and the expression levels of PD-L1 (CD274). We observed a negative correlation between the radiosensitivity index and the mRNA expression level of PD-L1 (**Supplementary Figure S4A**). Considering the entire cohort, PD-L1 status alone did not demonstrate a significant association with DSS or OS (**Supplementary Figure S4B**). Survival analysis was conducted for the four subgroups created based on a combination of radiosensitivity cluster and PD-L1 status in BRCA patients. Two groups were formed: the RS-PD-L1-high group and the other group. Subgroup analysis revealed that the RS-PD-L1-high group exhibited improved rates of DSS and OS, although the statistical significance for OS was not reached (**Figure 6A**). Utilizing the ESTIMATE algorithm, we observed increased estimate scores, stromal scores, and immune scores in the RS-PD-L1-high group compared to the other group, while tumor purity was found to be decreased (**Figure 6B**). Additionally, analysis using CIBERSORT demonstrated higher proportions of cytotoxic immune cells, including CD8 T cells, CD4 T cells, and NK cells, within the RS-PD-L1-high group (**Supplementary Figure S4C**). Analysis using the TIDE algorithm demonstrated a significant reduction in TIDE scores and T cell exclusion scores within the RS-PD-L1-high group compared to all other groups (**Figure 6C**). Notably, BRCA patients within the RS-PD-L1-high group exhibited a significantly improved response to immunotherapy compared to other groups (**Figure 6D**), further supporting the association between high PD-L1 expression and favorable immunotherapy outcomes in this specific patient population.

### 3.6 The association between the radiosensitivity model and chemotherapy and targeted therapy

**Figure 7:**
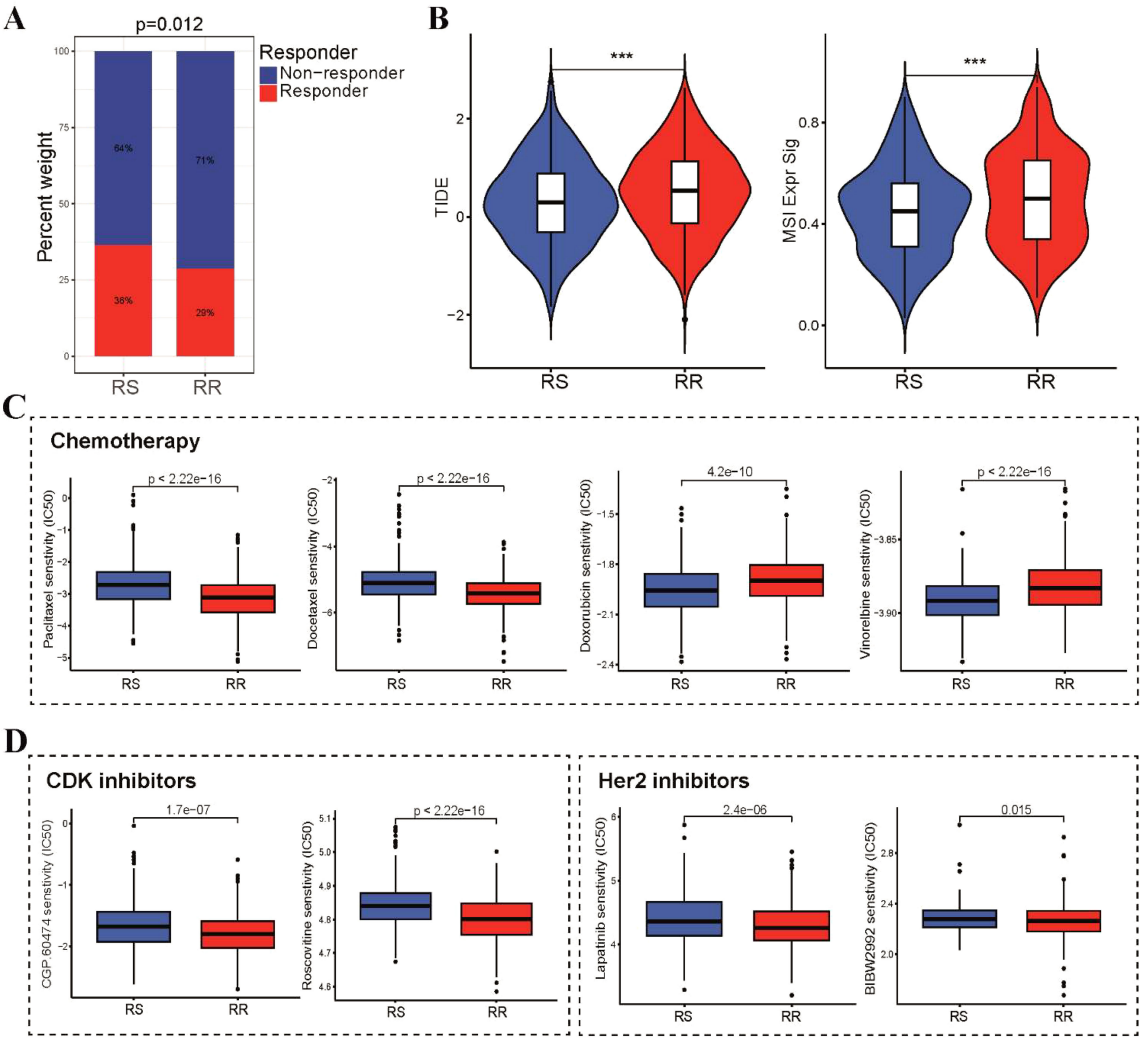
Differential sensitivity to immunotherapy, chemotherapy and targeted therapy between RS and RR groups. **(A)** A stacked histogram depicting the proportions of responder and non-responder patients was used to differentiate the two groups. **(B)** TIDE, and MSI scores were calculated and compared between the RS and RR groups. **(C)** Box plots illustrating the IC50 values of three first-line chemotherapeutic drugs (paclitaxel, docetaxel, doxorubicin and vinorelbine) for BRCA in the two groups. **(D)** Difference in IC50 values of CDK and Her2 inhibitors for BRCA in the two groups. ***p<0.001.

We subsequently investigated the correlation between the radiosensitivity model and the efficacy of immunotherapy, chemotherapy and targeted therapy in patients with BRCA. Notably, patients in the RS group demonstrated a higher likelihood of benefiting from administration of immune checkpoint inhibitors (ICI) (**Figure 7A**). This observation was further supported by the TIDE analysis results, which revealed lower TIDE scores, T cell exclusion and T cell dysfunction scores in the RS group compared to the RR group (**Figure 7B**). In addition, we investigated whether these two groups could have varying responses to first-line chemotherapeutics and targeted drugs. To achieve this, we utilized the pRRophetic R package, which leverages the GDSC pharmacogenomic database to predict drug sensitivity. Our findings highlight a differential sensitivity to chemotherapy agents between the RS and RR groups. The RS group exhibited significantly higher IC50 values for paclitaxel and docetaxel. Conversely, the RS group displayed lower IC50 values for doxorubicin and vinorelbine, indicating a potential enhanced sensitivity to these DNA-damaging drugs (**Figure 7C**). Furthermore, we investigated the sensitivity of the RR group to CDK and HER2 inhibitors by estimating their IC50 values. Our analysis revealed significantly lower IC50 values for multiple CDK and HER2 inhibitors in the RR group compared to other groups (**Figure 7D**). This finding suggests that these inhibitors may have the potential to enhance the radiosensitivity of the RR group, potentially by modulating cell cycle progression and/or targeting HER2-mediated signaling pathways.

### 3.7 *In vitro* experiments for validating the radiosensitivity genes

**Figure 8:**
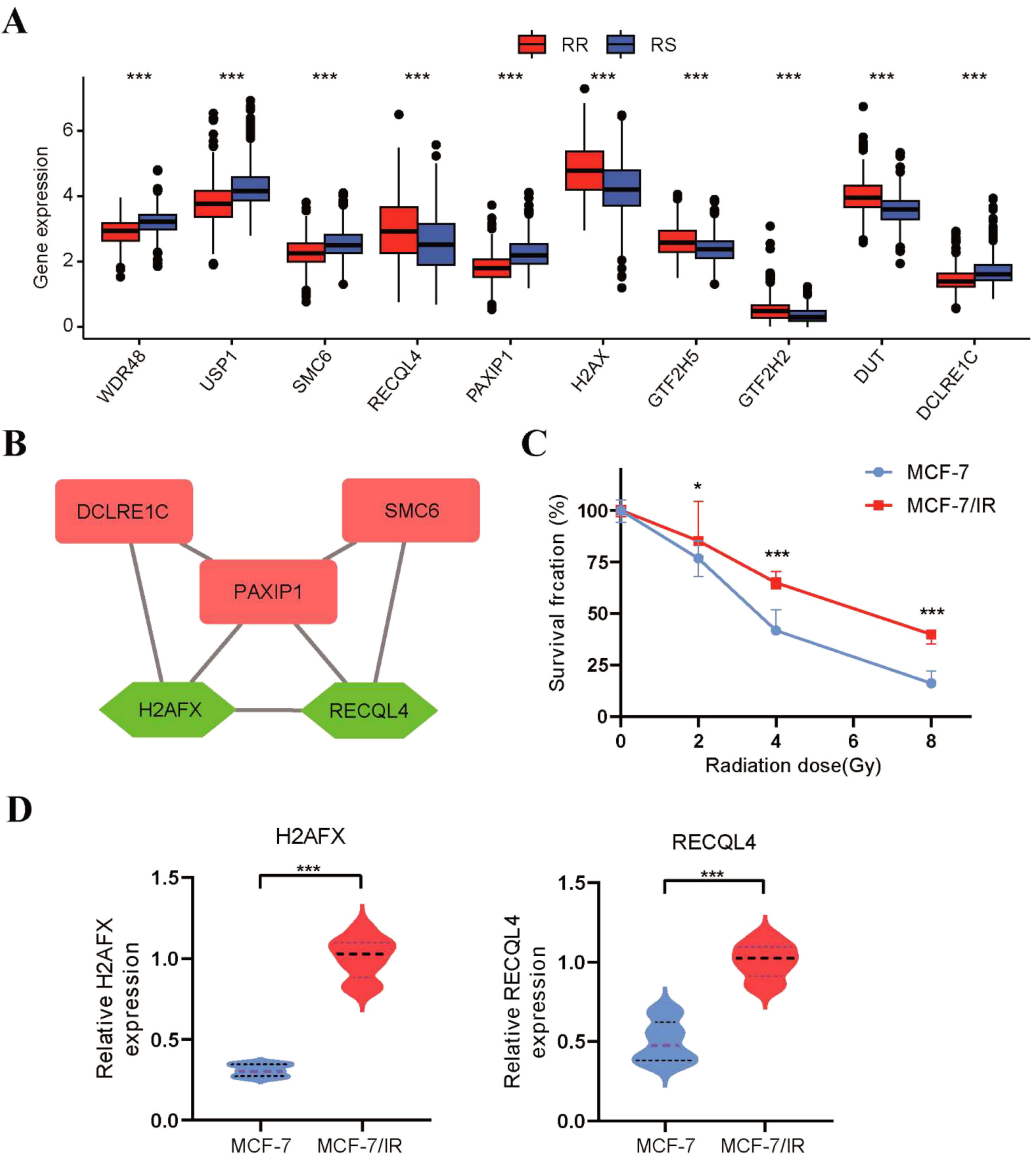
Validation of the radiosensitivity index through *in vitro* experiments. **(A)** The boxplot depicting the expression levels of 10 radiosensitivity genes between the RS and RR groups. **(B)** The top 5 genes with the highest MCC score were identified as hub genes in the PPI network. **(C)** The proliferative capacity of MCF-7 and MCF-7/IR cells after radiation exposure was evaluated using the CCK8 assay. **(D)** The qRT-PCR was utilized to assess the expression levels of H2AFX and RECQL4 in MCF-7 and MCF-7/IR cells. *p<0.05, **p<0.01, ***p<0.001.

To strengthen the validation of the radiosensitivity model’s predictive capability, we conducted *in vitro* experiments to confirm the expression of a 10-gene radiosensitivity index. The expression level of 10 genes in radiosensitivity model between the RS and RR groups were displayed in **Figure 8A**. Subsequently, we constructed a PPI network of these 10 genes using the STRING database and visualized it in Cytoscape software. This network consisted of 9 nodes and 9 edges, as shown in **Supplementary Figure S5**. Additionally, we employed the MCC algorithm of the cytohubba plugin to select hub genes from the PPI network. **Figure 8B** presented the hub genes identified based on their MCC scores. The five genes with the highest scores, namely PAXIP1, SMC6, H2AFX, DCLRE1C, and RECQL4, were selected as the hub genes for further validation. To verify the expression of these five hub genes, we utilized non-radioresistant (MCF-7) and radioresistant (MCF-7/IR) cells. The proliferative capacity of these cells after radiation exposure was assessed using the CCK-8 assay. In comparison to MCF-7 cells, the viability of MCF-7/IR cells demonstrated an increase upon exposure to radiation doses of 2, 4, and 8Gy, indicating the radioresistant ability of MCF-7/IR cells (**Figure 8C**). As displayed in **Figure 8D**, the expression levels of H2AFX and RECQL4 were higher in the radioresistant MCF-7/IR cells than in the non-radioresistant MCF-7 cells, consistent with our bioinformatics analysis. However, we did not observe any statistically significant differences in the expression levels of PAXIP1, SMC6, and DCLRE1C between the RS and RR groups.

## 4 Discussion

The effectiveness of radiation therapy in treating cancer relies on its ability to induce lethal DNA damage in cancer cells (45). Therefore, exploring the DNA damage repair pathway can provide valuable insights into potential biomarkers and targets for interference in BRCA radiotherapy. In this study, we aimed to develop a radiosensitivity model that can predict the radiosensitivity of BRCA patients based on the expression profiles of DNA repair-related genes (46). Through our analysis, we identified 10 novel DNA repair-related genes that were previously unreported in association with radiation sensitivity. We also demonstrated that perturbation of these genes is sufficient to induce alterations in the response to radiation. Additionally, we investigated the relationship between radiosensitivity and PD-L1 status, immune status in BRCA patients undergoing radiotherapy. Overall, our study contributes to the understanding of radiotherapy in BRCA and may have implications for future efforts to personalize treatment approaches and incorporate other therapeutic modalities.

The establishment of a predictive model for radiosensitivity would greatly facilitate the advancement of personalized cancer treatment (47). In this study, we observed that the RS group exhibited superior OS and DFS rate compared to the RR group when patients were received radiotherapy. Notably, this difference was not observed among patients who did not undergo radiotherapy. With the advancements in high-throughput sequencing, researchers have constructed gene signatures that can predict the radiosensitivity of patients with BRCA. Tang et al. successfully devised and validated immune-related and hypoxia-related gene signatures that effectively predicted radiosensitivity in BRCA patients (15). Moreover, a six-gene-based signature was established as a predictive model for estimating the sensitivity of BRCA to radiotherapy (48). However, it is important to note that the term “radiosensitivity” encompasses varying definitions dependent on its contextual application. In the context of clinical practice, radiosensitivity is delineated based on a minimum of two criteria. Firstly, the RS group’s survival rate should not surpass that of the RR group in the absence of radiotherapy. Secondly, when both groups undergo radiotherapy, the RS group should demonstrate significantly greater survival benefits compared to the RR group (49). Our findings suggest that the RSI has the potential to serve as a predictive biomarker for radiotherapy response in breast cancer. By stratifying patients based on their RSI scores, clinicians may be able to better personalize treatment strategies, avoiding unnecessary toxicity in patients who are unlikely to benefit from radiotherapy alone and intensifying treatment for those at higher risk of recurrence.

We proceeded to examine the relationships between clinicopathological factors, molecular subtype and radiosensitivity in our study. Our study revealed a higher incidence of ER/PR-positive and HER2-positive tumors in the RR group. This correlation between ER/PR status and radiosensitivity has also been reported in a previous study conducted by Javier et al. and Ah Kim et al.’s teams (50, 51). They found that ER-positive or PR-positive status was more commonly observed in the RR group of BRCA patients. Furthermore, in terms of molecular subtypes, the RR group exhibited a higher prevalence of normal-like and HER2-positive tumors, whereas the RS group had a higher frequency of the basal subtype. These findings were consistent with previous studies indicating that triple-negative BRCA subtypes are more predominant in the RS group (50). One potential explanation for these findings could be that the activity of the ER pathway is associated with the immune response to tumor cell death, which may be positively correlated with radiosensitivity (52). In clinical practice, the RSI could be readily integrated into existing diagnostic workflows. Upon diagnosis, a patient’s tumor sample could be analyzed to determine their RSI score. This information, combined with other clinicopathological factors, could then be used to guide treatment decisions.

In our analysis of the mutational profiles of the 10 DNA repair genes within the radiosensitivity index, we observed a significant prevalence of mutations, particularly missense mutations, in key genes such as PIK3CA and TP53. These mutations are known to impair the DNA damage response, potentially leading to increased genomic instability and altered cellular responses to radiation therapy (53, 54). Additionally, the presence of CNVs revealed that genes like RECQL4 and DCLRE1C exhibited amplifications, while others, including H2AX and GTF2H5, showed significant deletions. These CNV alterations may contribute to the abnormal expression patterns observed in our study, further influencing the radiosensitivity of tumors. Understanding these mutational landscapes is crucial, as they not only provide insights into the biological mechanisms underlying radiosensitivity but also highlight potential biomarkers for predicting patient responses to radiotherapy. These genes may specifically influence radiotherapy outcomes due to their roles in the DNA damage response mechanisms activated by radiation. Our findings underscore the importance of integrating mutational profiles into the assessment of therapeutic strategies for BRCA patients, paving the way for personalized treatment approaches.

It is well-established that radiotherapy can elicit an immune response within the tumor microenvironment (55). In our study, we initially explored the association between radiosensitivity and immunological factors, such as the expression of PD-L1 and ESTIMATE immune scores. Previous research has shown that PD-L1 expression influences the efficacy of radiotherapy, as it promotes PD-L1 expression and inhibits the function of cytotoxic lymphocytes (56, 57). Contrary to our expectations, we discovered that patients with high PD-L1 expression were more prevalent in the RS group. And BRCA patients in the RS-PD-L1-high group may have greater immune infiltration levels and higher ESTIMATE immune scores compared to other groups. This aligns with existing literature demonstrating that increased PD-L1 expression is often associated with enhanced immune infiltration and response to radiotherapy in various cancers (58). Additionally, we observed improved DSS and OS rates in the RS-PD-L1-high group compared to the other groups. The higher survival rate in the RS-PD-L1-high group may be attributed to the presence of abundant immune cell infiltrates, enabling the tumors to acquire mechanisms that increase their sensitivity to radiation. As predicted, our analysis revealed heightened infiltration of lymphocytes, including CD8 T cells, activated NK cells, follicular helper T cells, and CD4 T cells, in the RS-PD-L1-high group compared to other groups. These immune cell profiles may have implications for radiosensitivity and immunotherapy response. Recent study has highlighted the importance of CD8 T cell infiltration in tumor response following radiotherapy (59). This finding not only supports the better survival outcome in the RS-PD-L1-high group but also suggests the potential for combination strategies involving immune checkpoint blockades. As anticipated, we also noted that patients in the RS group exhibited a greater likelihood of experiencing benefits from the use of immune checkpoint inhibitors in our study.

Tumor profiling has been instrumental in tailoring treatment strategies for various cancers (60, 61). While immunotherapy has shown promise in treating solid tumors, its use in BRCA is currently limited to triple-negative BRCA (62). Previous research by Dai et al. demonstrated the predictive value of a 10-gene-based radiosensitivity index in determining the therapeutic efficacy of immunotherapy across multiple cancer types (19). In our study, we observed that patients in the RS group, especially those belonging to the RS-PD-L1-high subgroup, exhibited a notable augmentation of anti-tumor immunity. Consistent with expectations, the RS group displayed a higher probability of deriving therapeutic benefits from immunotherapy administration. The heightened responsiveness of the RS-PD-L1-high subgroup may be attributed to several factors, including radiotherapy-induced immunogenic cell death and the restoration of T cell activity through PD-L1 blockade. Increased infiltration of immune cells suggests that the tumor microenvironment in this subgroup is particularly conducive to immunotherapy. Clinically, a combination of radiotherapy and PD-1/PD-L1 inhibitors may be effective for RS-PD-L1-high patients, and our radiosensitivity index could help identify those most likely to benefit. Future clinical trials should evaluate this approach in RS-PD-L1-high breast cancer.

Taxanes such as docetaxel and paclitaxel have been shown to disrupt microtubule function and induce cell cycle arrest at the G2/M phase, rendering them effective agents for sensitizing cancer cells to radiotherapy (63). In BRCA, the concurrent administration of paclitaxel in combination with radiotherapy was determined to exhibit notable efficacy and favorable tolerability in the treatment of BRCA (64). CDK4/6 inhibitors, including palbociclib, abemaciclib, and ribociclib, have received clinical approval for the treatment of BRCA. *In vitro* studies have demonstrated their capability to augment the radiosensitivity of ER-positive BRCA cells (65). Our study revealed that patients in the RR group exhibited heightened sensitivity to first-line chemotherapy regimens, including paclitaxel, docetaxel, camptothecin, and CDK inhibitors. It is common practice to employ a combination of chemotherapeutics, targeted drugs, and CDK inhibitors to optimize the anticancer effects of radiotherapy in BRCA treatment. Based on our findings, it is reasonable to speculate that combining radiotherapy with chemotherapy or CDK inhibitors may serve as an effective approach to enhance radiosensitivity and overcome radio-resistance in the RR group of patients.

Several limitations should be taken into account. Firstly, the research data utilized in our study were derived from public databases like TCGA and METABRIC, which have not been validated in clinical trials. This reliance on retrospective data introduces potential biases, including variations in follow-up information and treatment protocols, as well as biases specific to the TCGA dataset, limiting the generalizability of our findings. Secondly, the inherent heterogeneity of BRCA poses challenges to the reproducibility and universality of our results, as technical disparities and cross-platform comparisons may affect the robustness of our findings. Lastly, while previous studies typically employed immunohistochemistry to measure PD-L1 expression, we used CD274 mRNA expression as a surrogate marker. Although this approach is practical, it should be noted that discrepancies can arise between mRNA levels and protein expression, indicating a need for further validation.

## 5 Conclusions

In conclusion, predicting radiosensitivity is clinically valuable in radiotherapy. Our radiosensitivity index, incorporating DNA repair genes, accurately predicts radiosensitivity in BRCA, potentially enabling personalized radiotherapy by optimizing patient selection and minimizing toxicity. We observed more ER/PR-positive and HER2-positive tumors in the radioresistant group. Notably, the RS-PD-L1-high subgroup showed enhanced anti-tumor immunity and immunotherapy sensitivity. Future clinical trials should prospectively validate these findings and explore mechanisms driving immunotherapy response in this subgroup, including PD-L1 inhibitor combinations, to improve outcomes.

## Data Availability

The original contributions presented in the study are included in the article/supplementary material. Further inquiries can be directed to the corresponding authors.
