# Supplementary material for: Discovery of a DNA repair-associated radiosensitivity index for predicting radiotherapy efficacy in breast cancer
Source: Front Oncol. 2025 Mar 25;15:1439516. doi: 10.3389/fonc.2025.1439516 (PMC11975882; doi:10.3389/fonc.2025.1439516)
Supplement: Supplementary file 1 [file DataSheet1.docx]

**Supplement Figures**


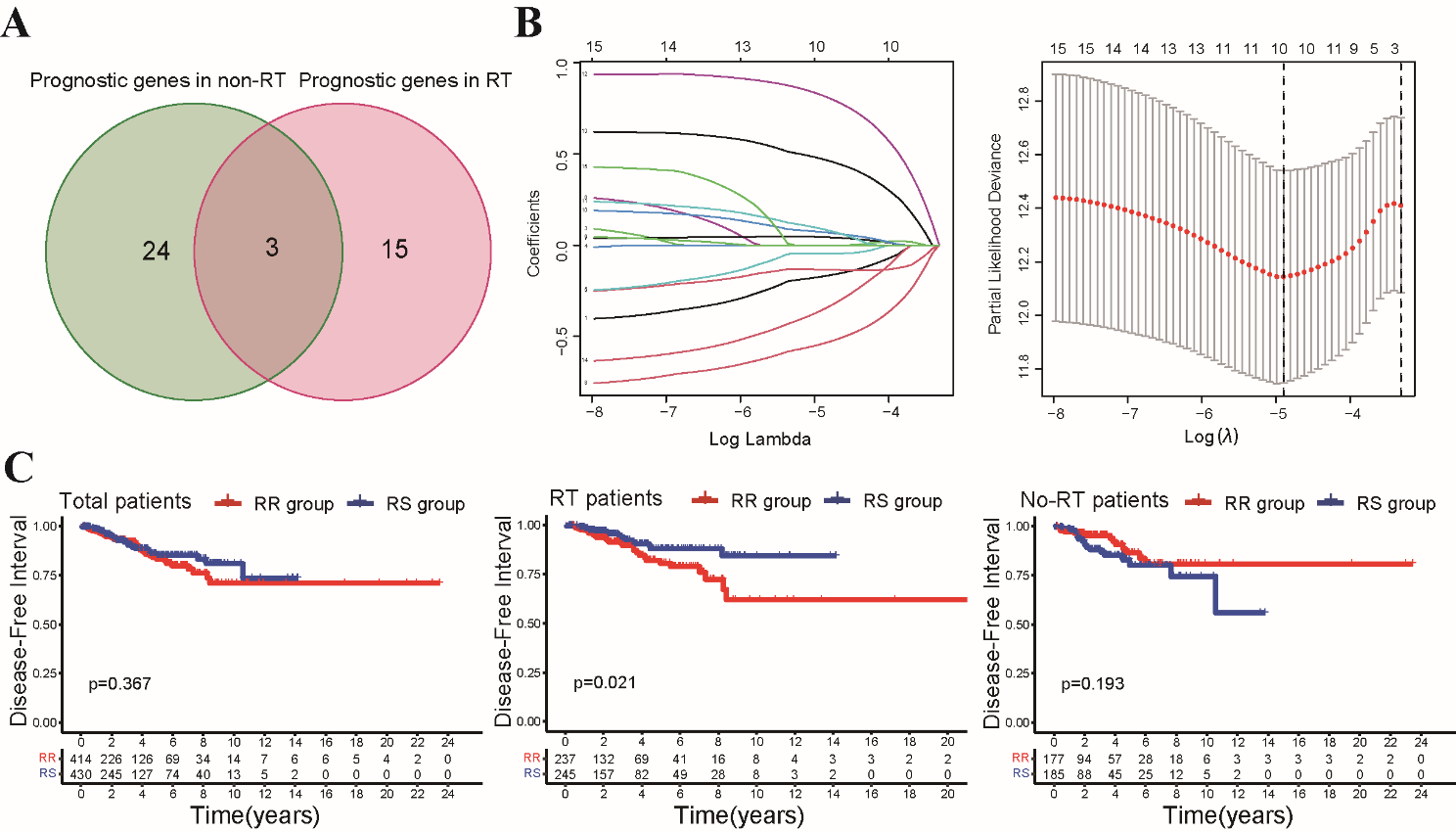


**FIGURE S1** **(A)** LASSO analysis identified 10 genes most associated with OS. **(B)** LASSO analysis identified 10 genes most associated with OS. **(C)** Kaplan-Meier survival curves showing the DFI outcomes of patients in the RS and RR groups within the radiotherapy patient subset. No significant difference in DFI rates was observed between the RS and RR groups in the non-radiotherapy patient subset or the entire patient cohort.


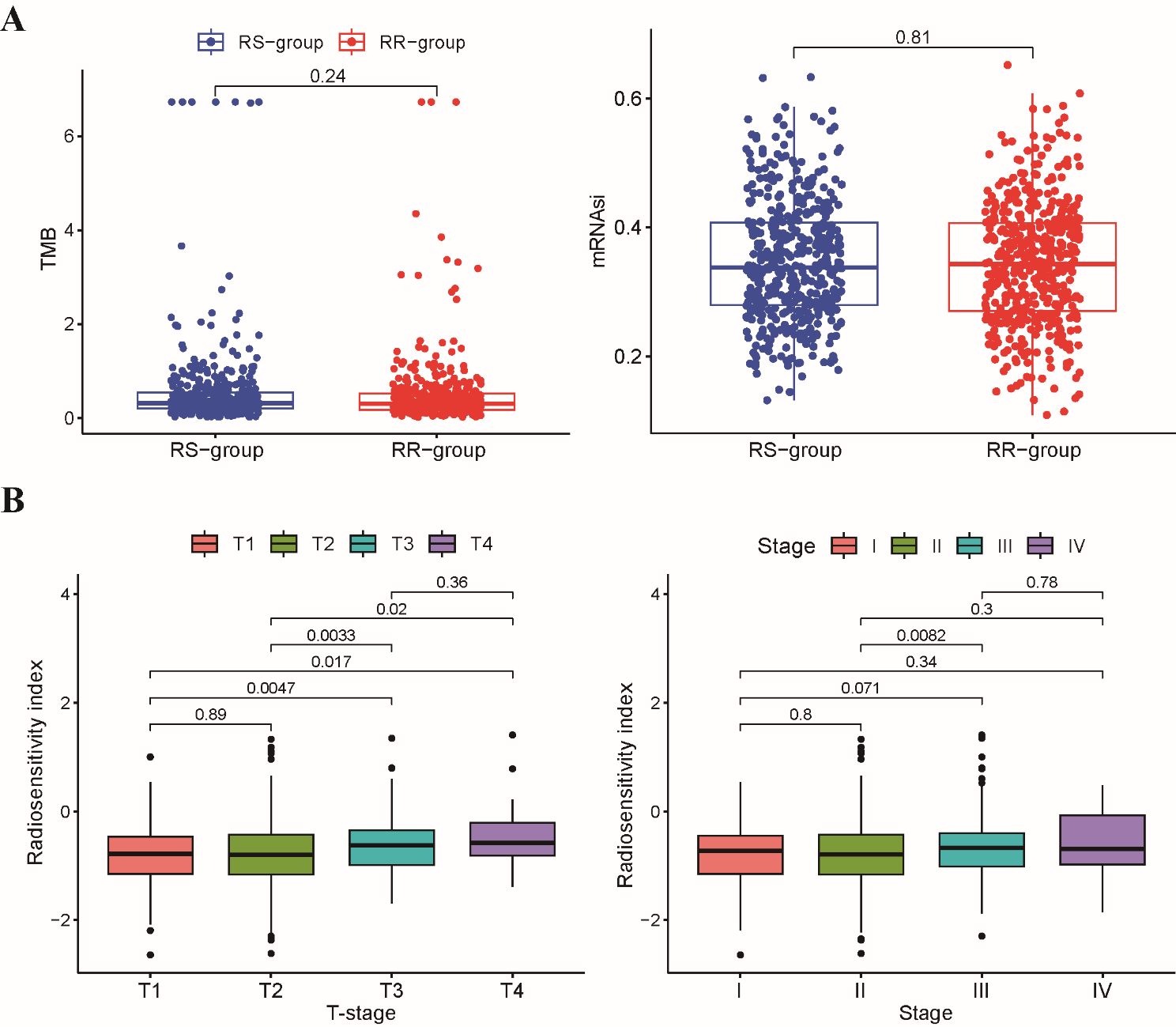


**FIGURE S2** **(A)** Comparisons of mRNAsi scores and TMB values between the RS group and RR group. **(B)** The scatter plot showed the correlation between the radiosensitivity index and tumor clinical stages.

**
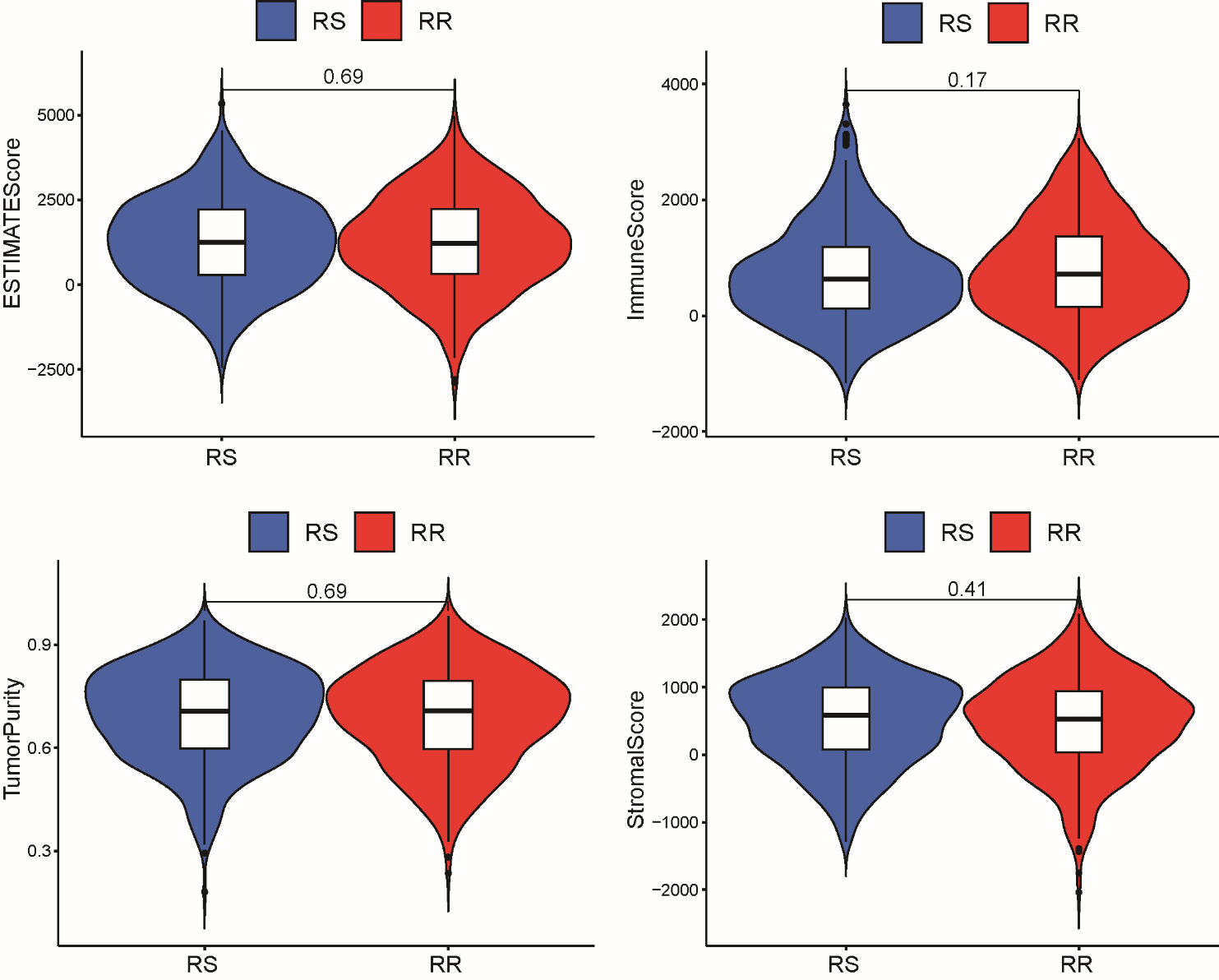
 FIGURE S3** The levels of infiltration for estimate scores, stromal scores, immune scores, and tumor purity between RS and RR groups were compared using violin plot.


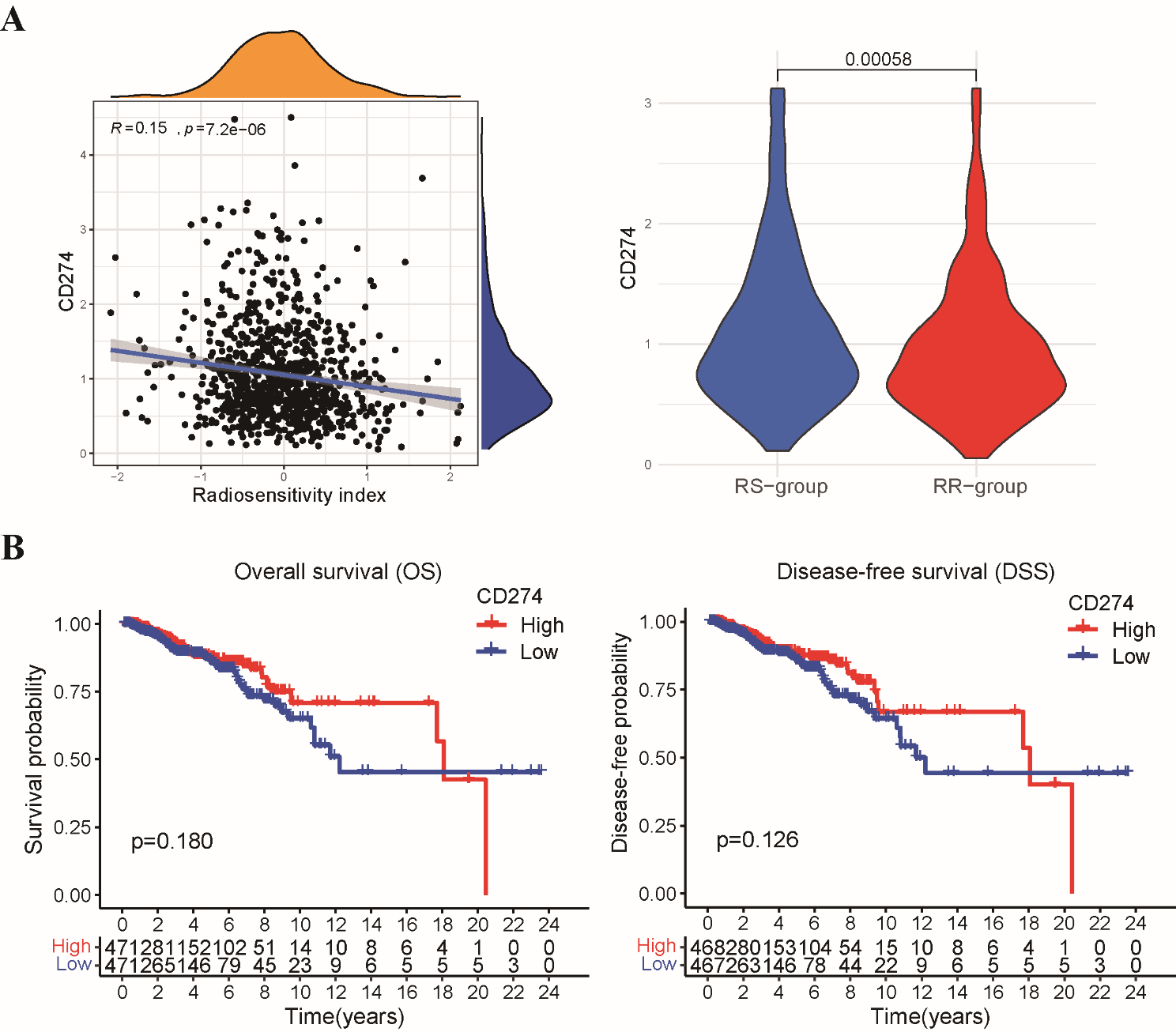


**FIGURE S4** **(A)** The expression levels of PD-L1 mRNA between the RS and RR groups, and correlation analysis between the radiosensitivity index and PD-L1 expression. **(B)** The Kaplan-Meier survival curves of DSS and OS between the high PDL1 and low PDL1 groups. **(C)** Proportions of 22 immune-infiltrating cells were compared between the RS-PD-L1-high and other groups. * p<0.05, ** p<0.01, *** p<0.001.

**TABLE S1 |** Sequences of the primer used for qRT-PCR

| mRNA | Forward primer | Reverse primer |
| --- | --- | --- |
| H2AX | CGGCAGTGCTGGAGTACCTCA | AGCTCCTCGTCGTTGCGGATG |
| RECQL4 | AAGCAACGGGAATCTGTCCTGC | CAGGCAAAAGCAACTGGAGGCA |
| PAXIP1 | CCAGGAGGAAAGCCATGTTCAC | CAGATGAGGACTGTGTTGCTGC |
| SMC6 | TGAAAGGGTCCTTCAGGCACTC | GGAAAGTCTGGATGATAAGCAGC |
| DCLRE1C | GAGCTAGAACAGTTCACCGAGAC | CAGGCTGCTTTTCTGATACTGCA |
| GAPDH | GTCTCCTCTGACTTCAACAGCG | ACCACCCTGTTGCTGTAGCCAA |

**TABLE S2 |** The formula for RSI and coefficient of 10 DRGs for the construction of radiosensitivity index

| Gene | Coef |
| --- | --- |
| WDR48 | -0.172264120215254 |
| USP1 | -0.134106777336494 |
| SMC6 | -0.0475356821453867 |
| RECQL4 | 0.0442865161311591 |
| PAXIP1 | -0.541363622807655 |
| H2AX | 0.0716730164617286 |
| GTF2H5 | 0.0886987290737661 |
| GTF2H2 | 0.821405301017306 |
| DUT | 0.472373540522277 |
| DCLRE1C | -0.356087842592145 |

**TABLE S3** Association between radiosensitivity group and clinicopathological characteristics.

| Covariates | Type | Total | RR | RS | P value |
| --- | --- | --- | --- | --- | --- |
| Age | <=50 | 297(31.53%) | 145(31.39%) | 152(31.67%) | 0.9818 |
|  | >50 | 645(68.47%) | 317(68.61%) | 328(68.33%) |  |
| Radiation | NO | 400(42.46%) | 191(41.34%) | 209(43.54%) | 0.5373 |
|  | YES | 542(57.54%) | 271(58.66%) | 271(56.46%) |  |
| Menopause | Peri | 36(3.82%) | 18(3.9%) | 18(3.75%) | 0.8647 |
|  | Post | 606(64.33%) | 293(63.42%) | 313(65.21%) |  |
|  | Pre | 211(22.4%) | 98(21.21%) | 113(23.54%) |  |
|  | unknow | 89(9.45%) | 53(11.47%) | 36(7.5%) |  |
| Clinical stage | stageⅠ-Ⅱ | 697(73.99%) | 330(71.43%) | 367(76.46%) | 0.0389 |
|  | stageⅢ-Ⅳ | 225(23.89%) | 125(27.06%) | 100(20.83%) |  |
|  | unknow | 20(2.12%) | 7(1.52%) | 13(2.71%) |  |
| T stage | T1-2 | 786(83.44%) | 373(80.74%) | 413(86.04%) | 0.0412 |
|  | T3-4 | 153(16.24%) | 87(18.83%) | 66(13.75%) |  |
|  | unknow | 3(0.32%) | 2(0.43%) | 1(0.21%) |  |
| N stage | N+ | 478(50.74%) | 242(52.38%) | 236(49.17%) | 0.3307 |
|  | N0 | 449(47.66%) | 212(45.89%) | 237(49.38%) |  |
|  | unknow | 15(1.59%) | 8(1.73%) | 7(1.46%) |  |
| M stage | M0 | 778(82.59%) | 365(79%) | 413(86.04%) | 0.467 |
|  | M1 | 17(1.8%) | 10(2.16%) | 7(1.46%) |  |
|  | unknow | 147(15.61%) | 87(18.83%) | 60(12.5%) |  |
